# Dizziness in older persons at high risk of future hospitalization: prevalence, differences between those with and without dizziness, and effect of a proactive primary care intervention

**DOI:** 10.1186/s12877-022-02910-1

**Published:** 2022-04-10

**Authors:** Ann-Sofi Kammerlind, Anneli Peolsson, Maria M. Johansson

**Affiliations:** 1 Department of Health, Medicine and Caring Sciences, Physiotherapy, Linköping University, Linköping, Sweden; 2 Futurum, Region Jönköping County, Jönköping, Sweden; 3 Occupational and Environmental Medicine Center, Department of Health, Medicine and Caring Sciences, Clinical Medicine, Linköping University, Linköping, Sweden; 4 Activity and Health, Department of Health, Medicine and Caring Sciences, Linköping University, Linköping, Sweden; 5 Acute Internal Medicine and Geriatrics, Department of Health, Medicine and Caring Sciences, Linköping University, Linköping, Sweden

**Keywords:** Aged, Dizziness, Geriatric assessment, Hospitalization, Prevalence, Primary health care

## Abstract

**Background:**

Dizziness is a common reason for seeking care, and frequently affects older persons. The aims were to determine the prevalence of dizziness in older persons at high risk of hospitalization, to compare subjects with and without dizziness, and to examine the effects on dizziness of a proactive primary care intervention in comparison with conventional care after one year.

**Methods:**

Data were derived from a prospective multicentre clinical trial in persons aged 75 and older and at high risk of hospitalization. A baseline questionnaire included demographic data, use of aids, questions about everyday physical activity and exercise, pain (intensity, frequency, and duration), activities of daily living measured using the ADL Staircase, and health-related quality of life measured using the EQ-5D-3L vertical visual analogue scale. Both at baseline and after one year, subjects were asked about dizziness, and those with dizziness answered the Dizziness Handicap Inventory – Screening version. Subjects in the intervention group were evaluated by a primary care team and when needed proactive care plans were established. Groups were compared using the Mann Whitney U-test or chi-squared test.

**Results:**

Of the 779 subjects, 493 (63%) experienced dizziness. Persons with dizziness differed regarding sex, homecare service, aids, activities of daily living, health-related quality of life, physical activity, and pain. The intervention did not significantly reduce the level of dizziness.

**Conclusions:**

Dizziness is common in vulnerable older persons, and individuals with dizziness differ in several respects. Further studies are needed employing more dizziness-specific assessment and individually tailored interventions.

**Trial registration:**

ClinicalTrials.gov 170608, ID: NCT03180606.

## Background

To meet the needs of the growing population of older high-need and high-cost patients, more knowledge is needed, both about this diverse population, and about the effects of tailored health care programmes [1, 2], not least in primary care [3]. Dizziness frequently affects older persons in particular [4] and is a common reason for seeking primary care [5]. There is a great variance in dizziness prevalence, ranging between 25–75% in different studies of older populations [4]. The causal and explanatory mechanisms are multifactorial and include both degenerative processes and different disorders of the peripheral and central parts of the vestibular system [4, 5], as well as medications [6, 7], and factors like avoidance of movement, fear of falling and inactivity [8]. Dizziness also increases the risk of falls [9, 10], health care resource use and costs [11], as well as mortality [12, 13].

Dizziness has previously been found to be related to the use of mobility aids [8], activities of daily living (ADL) [14], health-related quality of life (HRQoL) [8, 15–17], physical activity [8, 17], and pain [18] in older persons who were primarily selected based on age in population-based studies. However, these factors have not been examined in relation to dizziness in older persons at high risk of future hospitalization. To our knowledge, dizziness in relation to type of housing and use of homecare service has not previously been studied at all.

The aims were (1) to determine the prevalence of dizziness in older persons at high risk of future hospitalization, (2) to compare individuals with and without dizziness regarding type of housing, homecare service, use of mobility aids, HRQoL, ADL, physical activity, and pain, and (3) to examine the effects on dizziness of comprehensive geriatric assessment and proactive primary care medical and social interventions in comparison with conventional care after one year.

## Methods

### Study design and participants

**Fig. 1:**
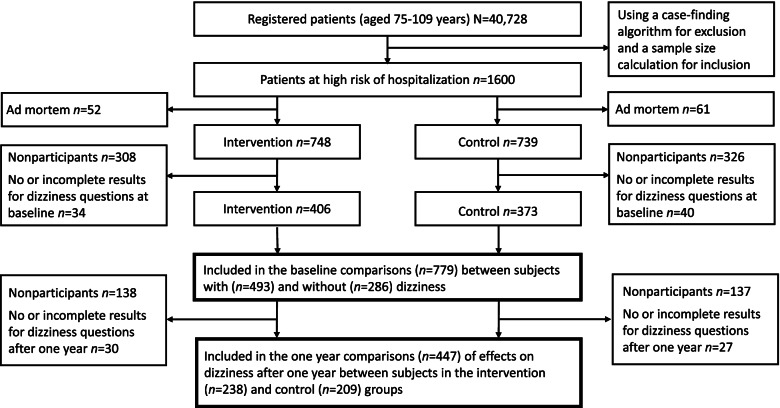
Flow chart outline of the inclusion of participants in the study and the analyses

In this study secondary outcome data about dizziness were derived from a prospective pragmatic multicentre clinical trial with the aim of evaluating the effects of a proactive primary care medical and social intervention in comparison with conventional care performed at 19 primary care practices (9 intervention practices and 10 control practices) in Sweden on frail persons aged 75 and older [19]. Subjects were selected by a statistical prediction model to find individuals at high risk of unplanned hospitalization within three months. The prediction model was based on previous research and the principal predictors were age, health care use, and diagnoses from inpatient care and outpatient visits over the preceding 12 months [20]. A cut off value (risk for hospital care) was calculated for each individual and depending on the preferred size of the predicted population the sensitivity, specificity and positive predictive value was altered [20]. The study took place in the county of Östergötland in Sweden, and out of all residents aged 75 and over (*N* = 40,728) a total sample size of 1600 individuals was calculated, and a postal questionnaire with an invitation letter was sent to 1487 persons who were still alive when the study started. Half of the subjects received the proactive intervention at selected primary care centres. The other half were controls and received care as usual in other similar primary care centres (in the same region, matched based on number of patients listed and urban/rural) whose staff were not aware of the control patients [19]. When needed, relatives or caregivers could participate and provide help with filling out the questionnaire. In total, 853 persons answered the questionnaire, and 779 had complete results for the questions about experiencing dizziness and were included in the present study (Fig. 1).

### Measures

The postal questionnaire at baseline included questions regarding age, sex, type of housing, use of homecare service, use of the mobility aids cane/crutch, walker, or wheelchair (transformed to the six categories shown in Table 2), and use of other aids. The following measurements were included in the same questionnaire.

ADL independence/dependence was assessed using the ADL Staircase [21] with ten ADL items (feeding, continence, transferring, going to the toilet, dressing, bathing, cooking, shopping, cleaning, transportation). Each item was scored on a three-point scale, giving zero (independent), one (partly dependent), and two (dependent) points, with a total score ranging from zero (independent in all activities) to 20 (dependent in all activities). Different aspects of reliability and validity have been studied and found to be good [22, 23].

HRQoL was measured using the European Quality of Life EQ-5D-3L vertical visual analogue scale (EQ-VAS) in the form of a thermometer ranging from zero (worst imaginable state of health) at the bottom to 100 (best imaginable state of health) at the top [24]. The Swedish version of EQ-VAS has shown good test–retest reliability (ICC (95% CI) 0.97 (0.93–0.99)) in individuals with dizziness and/or disequilibrium [25].

The results from two questions about physical activity, one about everyday physical activity and one about exercise/sport/open-air activity in a typical week during the previous 12 months, were combined into a four-point physical activity scale ranging from inactive to highly active [26].

Pain intensity over the previous week was measured using a 100 mm visual analogue scale (VAS), a reliable and valid scale to measure pain ranging from zero (no pain) to 100 (worst imaginable pain) [27]. Respondents were also asked to assess pain frequency on a five-point scale ranging from never to constantly, as well as pain duration in months.

Both at baseline and after one year, subjects were asked if they experienced dizziness (yes/no), and those with dizziness answered the Dizziness Handicap Inventory – Screening version (DHI-S) [28], which consists of ten questions measuring the impact of dizziness on handicap and quality of life. There were three response options for each item, giving four (yes), two (sometimes) and zero (no) points each, with a total score ranging from zero, suggesting no handicap, to 40, indicating a severe level of self-perceived handicap. The Swedish version of the scale has shown good test–retest reliability (ICC (95% CI) 0.91 (0.81–0.96)), and a method error of three points in individuals with dizziness and/or disequilibrium [29].

### Proactive primary care intervention

Subjects in the intervention group were approached by a primary care team (a registered nurse and a general practitioner), who evaluated social and medical conditions in a comprehensive geriatric assessment (CGA). The team used a CGA tool ´The Primary care Assessment Tool for the Elderly´(PASTEL) [30] constructed for the project as a help to get a holistic evaluation of the person and meet their individual needs [20]. The tool includes questions on dizziness and balance problems and other symptoms. Seventy-four percent of the subjects in the intervention group accepted the invitation to the CGA. All participants had a medication review. Based on the CGA evaluation the nurse-GP team established individual care plans when needed according to their clinical judgment. Examples of actions included initiation of homecare, diet counselling, physical activity advice, referrals to physiotherapist (PT) and/or occupational therapist (OT), and advice/referrals for were to get social support for loneliness and isolation [31]. A clinical follow-up by a nurse after one year were reported in 345 cases, and out of those 53 (15%) persons had been referred to a PT and 34 (10%) had been referred to an OT during that year. No data is available on the actions taken by PT and OT or how many sessions participants had with the PT or OT, and no extra rehabilitation resources came with the project. Subjects were not routinely screened for different dizziness diagnoses and did not routinely receive vestibular rehabilitation training or other specific instructions in managing dizziness.

### Statistical analysis

Data were reported as median with interquartile range (IQR), or as numbers with percentages. The intervention and control groups as well as the groups with and without dizziness were compared using the Mann Whitney U-test for scale data and chi-squared test for nominal or ordinal data. T-tests were also performed as an alternative to the Mann Whitney U-test and showed similar but unreported results. The ADL Staircase was analysed using the three-graded scale as recommended by Fänge et al. [32]. Age and sex distribution were compared with t-tests and chi-squared tests between the total population receiving the invitation letter (*n* = 1487) and the study samples at baseline (*n* = 779) and after one year (*n* = 447). Analyses were performed using IBM SPSS Statistics version 24. Because of the large sample size when comparing different factors between subjects with and without dizziness at baseline, the *p*-value for the analyses in Table 2 was adjusted to the stricter *p* < 0.001 to lower the risk of type 1 errors. But otherwise, statistical significance was defined as *p* ≤ 0.05.

## Results

The analytical sample consisted of 779 participants (369 (47%) women) with a mean age of 84 (range 75–104) years. Age and sex distribution did not differ between this study sample (*n* = 779) and the total population receiving the invitation letter (*n* = 1487). Background characteristics of the subjects are presented in Table 1. Of the 779 subjects, 493 (63%) experienced dizziness (Table 1).

**Table 1:** Background characteristics and prevalence of dizziness in the total study population (*n* = 779) at baseline

Age, years, mean (SD), min–max		84 (6), 75–104
Sex, *n* (%)	Women	369 (47)
	Men	410 (53)
Marital status, *n* (%) (*n* = 766)	Married/domestic partnership	381 (50)
	Unmarried	103 (13)
	Widow/widower	282 (37)
Type of housing, *n* (%) (*n* = 772)	Own house	260 (34)
	Apartment	451 (58)
	Serviced apartment	26 (3)
	Nursing home	29 (4)
	Dementia nursing home	6 (1)
Homecare service (*n* = 735)	Yes, daily	148 (20)
	Yes, but not daily	50 (7)
	No	537 (73)
Mobility aids, *n* (%) (*n* = 758)	None	280 (37)
	Only cane/crutch	67 (9)
	Only walker	189 (25)
	Cane/crutch and walker	135 (18)
	Wheelchair and cane/crutch/walker	64 (8)
	Only wheelchair	23 (3)
Dizziness, *n* (%)	Yes	493 (63)
	No	286 (37)

### Comparisons between the groups with and without dizziness

Persons with dizziness had a higher percentage of women (*p* < 0.001) compared to persons without dizziness, but age (*p* = 0.045) or type of housing (*p* = 0.001) did not differ significantly. Those with dizziness used homecare services (*p* < 0.001), aids in general (*p* < 0.001), and mobility aids (*p* < 0.001) to a higher degree. There was also a higher degree of ADL dependency (*p* < 0.001), a lower degree of HRQoL (*p* < 0.001), and physical activity (*p* < 0.001) in the group with dizziness. Pain intensity last week (*p* < 0.001) and pain frequency (*p* < 0.001) was higher among the individuals with dizziness, but pain duration did not differ (*p* = 0.243) (Table 2).

**Table 2:** Comparison of different factors between subjects with and without dizziness at baseline

		Dizziness (*n* = 493)	No dizziness (*n* = 286)	*P*-value^a^
Age, years, median (IQR) (*n* = 779)		84 (9)	83 (9)	.045^b^
Sex, *n* (%) (*n* = 779)	Women	263 (53)	106 (37)	** < .001**^c^
	Men	230 (47)	180 (63)	
Type of housing, *n* (%) (*n* = 772)	Own house	142 (29)	118 (41)	.001^c^
	Apartment	298 (60)	153 (54)	
	Serviced apartment	20 (4)	6 (2)	
	Nursing home	24 (5)	5 (2)	
	Dementia nursing home	5 (1)	1 (0)	
Homecare service, *n* (%) (*n* = 735)	Yes, daily	113 (25)	35 (13)	** < .001**^c^
	Yes, but not daily	35 (8)	15 (5)	
	No	311 (68)	226 (82)	
Total number of aids, median (IQR) (*n* = 758)		2 (2)	0 (0)	** < .001**^b^
Mobility aids, *n* (%) (*n* = 758)	None	113 (24)	167 (59)	** < .001**^c^
	Only cane/crutch	44 (9)	23 (8)	
	Only walker	147 (31)	42 (15)	
	Cane/crutch and walker	105 (22)	30 (11)	
	Wheelchair and cane/crutch/walker	51 (11)	13 (5)	
	Only wheelchair	17 (4)	6 (2)	
ADL Staircase, sum score of dependency, median (IQR) (*n* = 679)		5 (7)	1 (4)	** < .001**^b^
Health-related quality of life, mm EQ-VAS, median (IQR) (*n* = 711)		50 (25)	69 (30)	** < .001**^b^
Physical activity, sum score, *n* (%) (*n* = 687)	Inactivity	182 (42)	71 (28)	** < .001**^c^
	Low activity	147 (34)	94 (37)	
	Moderate activity	87 (20)	69 (27)	
	High activity	16 (4)	21 (8)	
Pain intensity last week, mm VAS, median (IQR) (*n* = 673)		45 (37)	32 (37)	** < .001**^b^
Pain frequency, *n* (%) (*n* = 735)	Never	30 (6)	41 (15)	** < .001**^c^
	Occasionally	143 (30)	112 (41)	
	Every day	177 (37)	82 (30)	
	Several times per day	43 (9)	18 (7)	
	Constantly	80 (17)	21 (8)	
Pain duration, months, median (IQR) (*n* = 546)		15 (28)	18 (26)	.243^b^

^a^Significant *p*-values are marked in bold

^b^Mann Whitney U-test

^c^Chi-squared test

*ADL* activities of daily living; *EQ-VAS* European Quality of life EQ-5D-3L vertical visual analogue scale; *IQR* interquartile range; *VAS* visual analogue scale

### The effects of a proactive primary care intervention on dizziness

The presence of dizziness did not differ at baseline or after one year between the subjects in the intervention (*n* = 238) and control (*n* = 209) groups answering the dizziness questions both at baseline and after one year. Age and sex distribution did not differ between this study sample (*n* = 447) and the total population receiving the invitation letter (*n* = 1487). Among those with dizziness, there was also no difference regarding the level of dizziness handicap measured by DHI-S at baseline or after one year between the intervention and control groups, and the decrease of dizziness after one year in the intervention group was small and not significant (*p* = 0.052) (Table 3).

**Table 3:** Dizziness results compared between the subjects in the intervention and control groups answering the dizziness questions both at baseline and after one year

Dizziness present or not (*n* = 447)		Intervention group (*n* = 238)	Control group (*n* = 209)	*P*-value
Baseline, *n* (%)	Yes	142 (60)	130 (62)	.301^a^
	No	96 (40)	79 (38)	
One-year follow-up, *n* (%)	Yes	138 (58)	128 (61)	.491 ^a^
	No	100 (42)	81 (39)	
Improved (yes to no), unchanged, or impaired (no to yes), *n* (%)	Improved	26 (11)	18 (9)	.565 ^a^
	Unchanged	190 (80)	175 (84)	
	Impaired	22 (9)	16 (8)	
DHI-S (*n* = 272) (only subjects with dizziness at baseline included)		Intervention group (*n* = 142)	Control group (*n* = 130)	*p*-value
Baseline, median (IQR)		18 (15)	18 (16)	.796 ^b^
One-year follow-up, median (IQR)		16 (20)	18 (16)	.165 ^b^
Change score, median (IQR)		-2 (10)	0 (10)	.052 ^b^

^a^Chi-squared test

^b^Mann Whitney U-test

*DHI-S* Dizziness Handicap Inventory – Screening version; *IQR* interquartile range

## Discussion

To the best of our knowledge, this is the first study focusing on the prevalence of dizziness as well as differences between those with and without dizziness and the effects of a proactive primary care intervention for older persons at high risk of future hospitalization. Compared to previous studies of the prevalence of dizziness in different older populations [4], a high proportion (67%) reporting dizziness was found in these older persons at high risk of future hospitalization. The dizziness group was not older than the non-dizziness group, probably because it is mainly other factors, such as diseases and drugs, rather than normal ageing, that explain the relationship between dizziness and age [33]. There was a higher percentage of women in the dizziness group in this study, and it is previously well established that dizziness is related to female sex [34].

Dizziness in relation to type of housing and use of homecare service has not to our knowledge been previously compared between individuals with and without dizziness. But the relationships found in this study were expected since dizziness is known to be a strong contributor to disability in the elderly [33], and dizziness has been reported to be common among older homecare recipients in Europe [35]. Persons with dizziness have been found to use mobility aids to a greater degree in a population-based study [8], and this, as well as a higher total number of aids, was confirmed in the present study in persons at high risk of future hospitalization. The present study also showed a greater degree of dependency in ADL, a lower health-related quality of life, and a lower degree of physical activity in persons with dizziness and high risk of future hospitalization, as has previously been seen in population-based studies including older persons selected on the basis of age [8, 14–17].

Pain intensity and pain frequency, but not pain duration, were higher in the individuals with dizziness in this study. The analyses of all these three aspects, not only the presence or absence of pain, in persons with and without dizziness, are unique and a strength of this study. Both pain and dizziness have been identified as strong contributors to poor self-rated health among vulnerable older persons [36]. Menant et al. [18] found that older community-dwelling persons with dizziness were more likely to report neck and back pain. Malmström et al. [37] reported that consecutive patients, referred to a vestibular or psychiatric unit who reported dizziness also more frequently reported pain. There are several possible mechanisms behind the relationship between dizziness and pain. Both symptoms might have the same cause, for example in migraine-associated headaches and vertigo/dizziness [38]. Pain might cause dizziness, for example in cervicogenic dizziness because of a disturbance of neck proprioception. Dizziness might also cause pain, for example due to inactivity and avoidance of head movements.

A postal questionnaire was used for the data collection, and some participants did not answer all questions. Therefore, there are some data missing for example in the pain, physical activity, and ADL variables, which is a limitation of the study.

The small reduced degree of dizziness seen after one year in the intervention group was not significant (*p* = 0.052), and only 2 points on DHI-S, which is below the method error of 3 points [29]. The proactive primary care intervention consisted of a comprehensive geriatric assessment that was standardized and given to all participants, but the following interventions were tailored and given according to the individual needs of the participants and based on the clinical judgement of the team. It is a limitation that the study was not randomized since there may be unknown differences between the practices. But the control group received usual care and did not come for any extra visits because of the study, which is a strength of the study. Information about specific actions given to the participants was not gathered, which is a limitation. Common actions included, for example, evaluation of medication and physical activity advice [19], and these interventions could be expected to reduce dizziness symptoms because dizziness is related to both use of many medications and some specific medications [6, 7] as well as inactivity [8]. Few participants received physical therapy so this was not one of the most common interventions, which is also a limitation of the study. Also, the actions taken within physical therapy were individual and a spectrum from one assessment with advice to a longer session of training individual or in a group might have been used. The effect on dizziness would probably have been greater if vestibular rehabilitation [39] and other physical therapy interventions had been given more extensively to the participants in this study.

Stam et al. [40] evaluated the effectiveness of a multifactorial intervention for dizziness in older people who were consulting their general practitioner about dizziness. The intervention consisted of medication adjustment in cases of fall-risk-increasing drugs and/or stepped mental health care in cases of anxiety/depression, and/or exercise therapy in cases of impaired functional mobility, and the control group received usual care. The results showed no significant intervention effect on dizziness, and adherence to the multifactorial intervention was low, especially in participants eligible for more than one intervention [40]. A qualitative study in primary care showed that older persons with chronic dizziness have needs that are not met by health care [41]. Parry et al. [42] found that unmet needs were targeted with a multidisciplinary and multifactorial assessment when fall risk, syncope, or dizziness were found in an initial screening. It is therefore important to always ask older vulnerable patients in primary care about dizziness, and to provide dizziness-specific assessment and individually tailored interventions based on the individual causes of dizziness in order to decrease the negative consequences of this frequent symptom.

## Conclusions

Dizziness is common in older persons who are judged to have a high risk of future hospitalization based on high age, health care use, and number of diagnoses over the preceding 12 months. Individuals with dizziness in our study differed regarding sex, use of homecare service and aids, ADL, HRQoL, physical activity, and pain compared to those without dizziness. A proactive primary care intervention did not significantly reduce the level of dizziness handicap after one year. Further studies are needed employing more dizziness-specific assessment and individually tailored interventions based on the individual causes of dizziness in vulnerable older dizziness patients in primary care.

## Data Availability

The datasets used and analysed during the current study are available from the corresponding author on reasonable request.

## Abbreviations

ADL: Activities of daily living
DHI-S: Dizziness Handicap Inventory – Screening version
EQ-VAS: European Quality of Life EQ-5D-3L vertical visual analogue scale
HRQoL: Health-related quality of life
IQR: Interquartile range
VAS: Visual analogue scale

## References

[1] Blumenthal D, Chernof B, Fulmer T, Lumpkin J, Selberg J (2016). Caring for high-need, high-cost patients – an urgent priority. N Engl J Med.

[2] Wammes JJG, van der Wees PJ, Tanke MAC, Westert GP, Jeurissen PPT. Systematic review of high-cost patients´ characteristics and healthcare utilisation. BMJ Open. 2018;8. 10.1136/bmjopen-2018-023113 PMC6129088 30196269

[3] Smeets RGM, Kroese M, Ruwaard D, Hameleers N, Elissen AMJ (2020). Person-centred and efficient care delivery for high-need, high-cost patients: primary care professionals´experiences. BMC Fam Pract.

[4] Brosel S, Strupp M (2019). The vestibular system and ageing. Subcell Biochem.

[5] Bösner S, Schwarm S, Grevenrath P, Schmidt L, Hörner K, Beidatsch D (2018). Prevalence, aetiologies and prognosis of the symptom dizziness in primary care – a systematic review. BMC Fam Pract.

[6] Lin E, Aligene K (2013). Pharmacology of balance and dizziness. NeuroRehabilitation.

[7] Shoair OA, Nyandege AN, Slattum PW (2011). Medication-related dizziness in the older adult. Otolaryngol Clin North Am.

[8] Kammerlind AS, Ernsth Bravell M, Fransson EI (2016). Prevalence of and factors related to mild and substantial dizziness in community-dwelling older adults: a cross-sectional study. BMC Geriatr.

[9] Dokuzlar O, Koc Okudur S, Smith L, Soysal P, Yavuz I, Aydin AE (2020). Assessment of factors that increase risk of falling in older women by four different clinical methods. Aging Clin Exp Res.

[10] Dokuzlar O, Koc Okudur S, Soysal P, Kocyigit SE, Yavuz I, Smith L (2020). Factors that increase risk of falling in older men according to four different clinical methods. Exp Aging Res.

[11] Wang X, Strobl R, Holle R, Seidl H, Peters A, Grill E (2019). Vertigo and dizziness cause considerable [sic] more health care resource use and costs: results from the KORA FF4 study. J Neurol.

[12] Corrales CE, Bhattacharyya N (2016). Dizziness and death: an imbalance in mortality. Laryngoscope.

[13] Juraschek SP, Longstreth WT, Lopez OL, Gottdiener JS, Lipsitz LA, Kuller LH (2020). Orthostatic hypotension, dizziness, neurology outcomes, and death in older adults. Neurology.

[14] Aggarwal  NT,  Bennett  DA, Bienias  JL, Mendes de Leon CF, Morris  MC, Evans  DA (2000). The prevalence of dizziness and its association with functional disability in a biracial community population. Gerontol A Biol Sci Med Sci.

[15] Lisko I, Tormakangas T, Jylha M. Structure of self-rated health among the oldest old: analyses in the total population and those living with dementia. SSM Popul Health. 2020;11. 10.1016/j.ssmph.2020.100567 PMC7110410 32258355

[16] Lindell E, Kollén L, Johansson M, Karlsson T, Rydén L, Falk Erhag H (2021). Benign paroxysmal positional vertigo, dizziness, and health-related quality of life among older adults in a population-based setting. Eur Arch Otorhinolaryngol.

[17] Kollén L, Hörder H, Möller C, Frändin K (2017). Physical functioning in older persons with dizziness: a population-based study. Aging Clin Exp Res.

[18] Menant JC, Wong A, Sturnieks DL, Close JCT, Delbaere K, Sachdev PS (2013). Pain and anxiety mediate the relationship between dizziness and falls in older people. J Am Geriatr Soc.

[19] Marcusson  J, Nord  M, Johansson  MM, Alwin  J, Levin L-Å, Dannapfel P (2019). Proactive healthcare for frail elderly persons: study protocol for a prospective controlled primary care intervention in Sweden. BMJ Open.

[20] Marcusson J, Nord M, Dong HJ, Lyth J (2020). Clinically useful prediction of hospital admissions in an older population. BMC Geriatr.

[21] Sonn U, Asberg KH (1991). Assessment of activities of daily living in the elderly: a study of a population of 76-year-olds in Gothenburg. Sweden Scand J Rehabil Med.

[22] Johansson MM, Barbero M, Peolsson A, Falla D, Cescon C, Folli A (2021). Pain characteristics and quality of life in older people at high risk of future hospitalization. Int J Environ Res Public Health.

[23] Sonn U (1996). Longitudinal studies of dependence in daily life activities among elderly persons. Scand J Rehabil Med Suppl.

[24] Brooks R (1996). EuroQol: the current state of play. Health Policy.

[25] Kammerlind AS, Bergquist Larsson P, Ledin T, Skargren EI (2005). Reliability of clinical tests and subjective ratings in dizziness and disequilibrium. Adv Physiother.

[26] Peolsson A, Almkvist C, Dahlberg C, Lindqvist S, Pettersson S (2007). Age- and sex-specific reference values of a test of neck muscle endurance. J Manipulative Physiol Ther.

[27] Williamson A, Hoggart B (2005). Pain: a review of three commonly used pain rating scales. J Clin Nurs.

[28] Jacobson GP, Calder JH (1998). A screening version of the Dizziness Handicap Inventory (DHI-S). Am J Otol.

[29] Kammerlind AS, Bladström M, Svensson K (2011). Test-retest reliability of two short Swedish versions of the Dizziness Handicap Inventory. Adv Physiother.

[30] Nord M, Östgren CG, Marcusson J, Johansson M (2020). Staff experiences of a new tool for comprehensive geriatric assessment in primary care (PASTEL): a focus group study. Scand J Prim Health Care.

[31] Nord M, Lyth J, Alwin J, Marcusson J (2021). Costs and effects of comprehensive geriatric assessment in primary care for older adults with high risk for hospitalisation. BMC Geriatr.

[32] Fänge A, Lanke J, Iwarsson S (2004). Statistical assessment of changes in ADL dependence: three-graded versus dichotomised scaling. Int J Rehabil Res.

[33] Mueller M, Strobl R, Jahn K, Linkohr B, Peters A, Grill E (2014). Burden of disability attributable to vertigo and dizziness in the aged: results from the KORA-Age study. Eur J Public Health.

[34] Neuhauser HK (2016). The epidemiology of dizziness and vertigo. Handb Clin Neurol.

[35] Stam H, van Vugt VA, Twisk JWR, Finne-Soveri H, Garms-Homolová V, Declercq A (2020). The prevalence and persistence of dizziness in older European home care recipients: a prospective cohort study. J Am Med Dir Assoc.

[36] Figueiredo S, Rosenzveig A, Morais JA, Mayo NE. Planning health services for seniors: can we use patient’s own perception? Can Geriatr J. 2017;20:66–74. 10.5770/cgj.20.248 PMC5495538 28690706

[37] Malmström EM, Magnusson M, Holmberg J, Karlberg M, Fransson P-A (2020). Dizziness and localized pain are often concurrent in patients with balance or psychological disorders. Scand J Pain.

[38] Teggi  R, Manfrin  M, Balzanelli  C, Gatti  O, Mura  F, Quaglieri S (2016). Point prevalence of vertigo and dizziness in a sample of 2672 subjects and correlation with headaches.. Acta Otorhinolaryngol Ital.

[39] Furman JM, Raz Y, Whitney SL (2010). Geriatric vestibulopathy assessment and management. Curr Opin Otolaryngol Head Neck Surg.

[40] Stam H, van der Wouden JC, Hugtenburg JG, Twisk JWR, van der Horst HE, Maarsingh OR. Effectiveness of a multifactorial intervention for dizziness in older people in primary care: a cluster randomised controlled trial. PLoS One. 2018;13. 10.1371/journal.pone.0204876 PMC6178383 30300371

[41] Olsson Möller U, Hansson EE, Ekdahl C, Midlöv P, Jakobsson U, Kristensson J (2014). Fighting for control in an unpredictable life: a qualitative study of older persons´ experiences of living with chronic dizziness. BMC Geriatr.

[42] Parry SW, Hill H, Lawson J, Lawson N, Green D, Trundle H (2016). A novel approach to proactive primary care-based case finding and multidisciplinary management of falls, syncope, and dizziness in a one-stop service: preliminary results. J Am Geriatr Soc.

